# Pulmonary hypertension secondary to hyperviscosity in a patient with rheumatoid arthritis and acquired von Willebrand disease: a case report

**DOI:** 10.1186/1752-1947-7-232

**Published:** 2013-10-02

**Authors:** Thierry Hernández-Gilsoul, Yemil Atisha-Fregoso, Angel G Vargas-Ruíz, Eduardo Rivero-Sigarroa, Guillermo Dominguez-Cherit, Silvio A Ñamendys-Silva

**Affiliations:** 1 Department of Critical Care Medicine, Instituto Nacional de Ciencias Médicas y Nutrición Salvador Zubirán, Mexico City, México; 2 Department of Immunology and Rheumatology, Instituto Nacional de Ciencias Médicas y Nutrición Salvador Zubirán, Mexico City, Mexico; 3 Department of Hematology/Oncology, Instituto Nacional de Ciencias Médicas y Nutrición Salvador Zubirán, Mexico City, Mexico; 4 Department of Critical Care Medicine, Instituto Nacional de Cancerología, Avenida San Fernando No. 22, Colonia Sección XVI Delegación Tlalpan, Mexico City, C.P. 14080 D.F. Mexico, Mexico

**Keywords:** Acquired von Willebrand disease, Hyperviscosity syndrome, Pulmonary arterial hypertension, Rheumatoid arthritis

## Abstract

**Introduction:**

Acquired von Willebrand disease is initiated by autoantibodies and hyperviscosity syndrome caused by a massive polyclonal hypergammaglobulinemia. Acquired von Willebrand disease associated with autoimmune disease in addition to pulmonary hypertension during emergency room presentation is a rare condition. To the best of our knowledge, this is the second case reported in the literature treated with success; the first one was reported in 1987.

**Case presentation:**

A 28-year-old mestizo man with a 3-year history of inflammatory arthritis was admitted to our hospital. An overlap of rheumatoid arthritis with systemic lupus erythematosus was suspected; therefore methotrexate was initiated, and later changed to leflunomide because of liver toxicity. Prothrombin time, international normalized ratio and activated partial thromboplastin times were normal (11/10.4 seconds; 1.2; 31.1/26.9 seconds, respectively), von Willebrand factor activity was observed with low ristocetin cofactor at 33.6UI/dL, high von Willebrand factor antigen >200UI/dL, and a low von Willebrand factor: ristocetin cofactor to von Willebrand factor antigen ratio. He was admitted to the emergency room with a 24-hour evolution of progressive dyspnea, cough, thoracic pain, and palpitations, 104 beats/min, 60/40 mmHg, temperature of 38°C, pulse oximetric saturation 88% and 30 breaths/minute. Cold, pale and mottled skin was also observed. He was then transferred to the intensive care unit. The placement of a pulmonary artery catheter was made. The initial patterns showed a precapillary pulmonary hypertension; acute pulmonary embolism was the first choice for diagnosis. Pulmonary angiography was conducted, and when no clot was discovered, pulmonary artery hypertension associated with connective tissue disease was considered. Serum protein electrophoresis confirmed the presence of a massive polyclonal hypergammaglobulinemia, and no paraproteinemia or monoclonal cell population was found from the electrophoretic pattern of the patient’s plasma. Hypergammaglobulinemia was the cause of hyperviscosity syndrome associated with autoantibodies. Three sessions of plasma exchange therapy were made, and clinical improvement was observed. He was then discharged from the intensive care unit and hospital, respectively. He is now attended by an external consult and has no respiratory symptomatology.

**Conclusions:**

Hyperviscosity syndrome with pulmonary arterial hypertension presentation in a patient with acquired von Willebrand disease in an autoimmune context is a rare condition that can be treated successfully with plasmapheresis and critical care support.

## Introduction

Acquired von Willebrand disease (aVWD) is initiated by autoantibodies and hyperviscosity syndrome (HVS) caused by a massive polyclonal hypergammaglobulinemia. AVWD associated with autoimmune disease in addition to pulmonary hypertension during emergency room (ER) presentation is a rare condition. Plasma cell neoplasm is the most common cause of HVS, mainly Waldenström macroglobulinemia and multiple myeloma, diseases characterized by a monoclonal paraprotein [1]. Three mechanisms for aVWD have been proposed: a) aggregation of immunoglobulins (Igs) dependent upon the factor region [2], b) interaction between IgM rheumatoid factor and intermediate complexes formed by IgG aggregates [3], and c) IgG intermediate complexes alone [4]. Viscosity refers to resistance to flow, or stickiness, and is measured by the time required for a serum or plasma sample to flow through a capillary tube (Ostwald tube) under the influence of gravity [5]. Sjögren syndrome, rheumatoid arthritis (RA), systemic lupus erythematosus (SLE), cryoglobulinemia, and human immunodeficiency virus infection are rarely associated with HVS [6].

According to the Scientific and Standardization Committee of the International Society of Thrombosis and Haemostasis registry, autoimmune disorders are the cause of aVWD in only 2% of cases [7]. RA diagnosis and the presence of antinuclear antibodies could be an epiphenomenon, which has been previously described [8]. However, the pathophysiology of aVWD is heterogeneous, and in most cases there is an increased clearance, inhibition or proteolysis of von Willebrand factor (VWF) in the plasma [9]. The first two mechanisms had been reported in SLE due to circulating IgG antibodies directed against either nonfunctional or functional domains of VWF [10].

In addition, it has been reported that during pulmonary hypertension, plasminogen activator inhibitor-1 (PAI-1) levels are high, suggesting a loss of fibrinolytic activity [11], however, PAI-1 was not determinate in our patient. The hemodynamic pattern has also been described by Condliffe *et al*. [12] who studied patients with connective tissue diseases and pulmonary arterial hypertension (PAH), as well as 28 patients with isolated SLE and PAH. Murata *et al*. [13] reported a case of PAH with Raynaud phenomenon and relative polycythemia where the right cardiac catheterization showed PAH with pulmonary artery pressures of 93/39mmHg (mean pressure 60mmHg), cardiac index of 2.2L/minute/m^2^, and a pulmonary vascular resistance index of 1219dyne·second/(cm^5^·m^2^). Patients with SLE and PAH not related with an incidence of hyperviscosity were found to have 1-year and 3-year survival rates of 78% and 74%, respectively, and patients with RA had 1-year and 3-year survival rates of 83% and 63%, respectively [12]. Here, we present a rare association of aVWD, PAH and RA. To the best of our knowledge, this is the second case reported in the literature treated with success. The first one was reported by Eaton and colleagues in 1987 [14]; they treated a patient with RA, serum hyperviscosity and secondary pulmonary hypertension, with a mean pulmonary arterial pressure of 53mmHg and pulmonary vascular resistance of 707dyne·second/cm^2^.

## Case presentation

A 28-year-old mestizo man with a 3-year history of inflammatory arthritis and dry mouth and eyes was admitted to our hospital. He was admitted to our hospital at the request of an external consultant in January 2008; the initial laboratory evaluation is listed in Table 1. Methotrexate was initiated with good response; 1 year later methotrexate was changed to leflunomide because of liver toxicity. Globulin levels at admission and 1 year after were 5.2 and 7.2g/dL, respectively. Of interest, although he also had positive anti-double-stranded deoxyribonucleic acid (DNA) and anti-nucleosomes antibodies, which normally have a higher specificity for the diagnosis of SLE, at admission we could not make a diagnosis because of the absence of clinical manifestations other than arthritis and sicca.

**Table 1:** Laboratory data at hospital: external consultant admission

**Test**	**Value**	**Normal value**
Anticitrulline antibodies	268U/mL	≤25U/mL
Antinuclear antibodies	Homogeneous pattern 1:5120 dilution Cytoplasmic pattern 1:160 dilution	Negative
Anti-double-stranded deoxyribonucleic acid	86.7U/mL	≤9.6U/mL
Anti-nucleosomes antibodies	128.4U/mL	≤71.9U/mL
C3	132mg/dL	52.8–170.9mg/dL
C4	67.2mg/dL	12.1–39.5mg/dL
SSA	3.5U/mL	≤10U/mL
SSB	4.6U/mL	≤10U/mL
Hemoglobin	11.4g/dL	13.5–18g/dL
Leucocytes	4900/μL	4000–10,000/μL
Platelets	220,000/μL	150,000–450,000/μL
Creatinine	0.6mg/dL	0.7–1.2mg/dL

Hyperemia and mild eye pain appeared in March 2009, and a diffuse scleritis was diagnosed. Therefore leflunomide was suspended and he received prednisone (1mg/kg), tapered to 5mg/day in 1 month and suspended after another month because a mouth ulcer appeared.

Multiple important mucosal bleeding episodes (epistaxis and gingival hemorrhage) started in 2009, and coagulation tests were conducted through 2010, with the following results: prothrombin time, international normalized ratio and activated partial thromboplastin times were normal (11/10.4 seconds; 1.2; 31.1/26.9 seconds, respectively), VWF activity was observed with low ristocetin cofactor (RCo) at 33.6UI/dL, high VWF antigen (VWF:Ag) >200UI/dL, and a low VWF:RCo to VWF:Ag ratio. Furthermore, an Ivy bleeding time test of more than 15 minutes and normal factor VIII activity (70UI/dL) were observed. An *in vitro* test showed that the patient’s IgG inhibited the VWF:RCo of normal plasma, therefore the RCo activity inhibition test was positive, although this was not necessary for the diagnosis. A characteristic aVWD laboratory test with the identification of a causal underlying disease, integrated the diagnosis [15].

The patient was admitted to the ER in April 2011 with a 24-hour evolution of progressive dyspnea, cough, thoracic pain, and palpitations, 104beats/minute, 60/40mmHg, temperature of 38°C, pulse oximetric saturation 88% and 30 breaths/minute. Cold, pale and mottled skin was also observed; laboratory values at ER admission are listed in Table 2. There were no signs of arthritis or bleeding. Initial crystalloid reanimation was made with partial response, and persistent tachypnea and hypoxemia (arterial oxygen tension/fraction of inspired oxygen = 245.7) precluded a rapid intubation sequence. He was then transferred to the intensive care unit (ICU) 12 hours later. Initial laboratory ER patient data are listed in Table 2; Acute Physiology and Chronic Health Evaluation II and Sequential Organ Failure Assessment values at ICU admission were 11 and 27 points respectively.

**Table 2:** Laboratory data at emergency room admission

**Arterial blood gas at room air**		
**pH 7.45, PaO**_**2**_**51.6mmHg, PaCO**_**2**_**14.7mmHg, HCO**_**3**_**10.6, standard BE -12.3mEq/L, SaO**_**2**_**85%**		
Hemoglobin	9g/dL	13.5–18g/dL
Leucocytes	14,900/μL	4000–10,000/μL
Platelets	121,000/μL	150,000–450,000/μL
Creatinine	1.62mg/dL	0.7–1.2mg/dL
BUN	23mg/dL	7–25mg/dL
Sodium	127mEq/L	137–145mEq/L
Potassium	4.7mEq/L	3.6–5mEq/L
Chloride	106mEq/L	98–110mEq/L
Total bilirubin	3.3mg/dL	0.2–1.3mg/dL
Indirect bilirubin	2.3mg/dL	≤0.3mg/dL
AST	147UI/L	5–35UI/L
ALT	244UI/L	7–56UI/L
Albumin	1.7g/dL	3.3–5g/dL
Globulin	7g/dL	2.6–4.6g/dL
α_1_-globulin	0.1g/dL	0.2–0.4g/dL
α_2_-globulin	0.4g/dL	0.6–1g/dL
β-globulin	0.3g/dL	0.6–1.2g/dL
γ-globulin	4.2g/dL	0.7–1.3g/dL
Immunoglobulin G	3702mg/dL	700–1600mg/dL
Immunoglobulin M	542mg/dL	40–230mg/dL
Immunoglobulin A	372mg/dL	70–400mg/dL

*Abbreviations*: *ALT* alanine aminotransferase, *AST* aspartate aminotransferase, *BE* base excess, *BUN* blood urea nitrogen, *HCO*_*3*_ bicarbonate, *PaCO*_*2*_ partial pressure of carbon dioxide in arterial blood, *PaO*_*2*_ partial pressure of oxygen in arterial blood, *SaO*_*2*_, saturation level of oxygen in hemoglobin.

An electrocardiogram showed an incomplete right bundle block not previously detected. A chest X-ray revealed no infiltrates, and complementary laboratory tests did not suggest a related infection. The placement of a pulmonary artery catheter (PAC) was made under the indication of a differentiation between cardiogenic and non-cardiogenic shock (see Table 3). The initial patterns showed a precapillary pulmonary hypertension; therefore, in the autoimmune context of the patient, although serial negative antiphospholipid antibodies were noted in his history, acute pulmonary embolism was the first choice for diagnosis. Pulmonary angiography was conducted, and when no clot was discovered, PAH associated with connective tissue disease was considered because pulmonary hypertension has been historically associated with connective tissue diseases.

**Table 3:** Pulmonary artery catheter patterns

**Variable**	**Admission**	**Day 2**	**Day 2 Dobutamine 3μg/kg/minute**	**Day 2 Dobutamine 6μg/kg/minute**	**Day 2 Dobutamine 9μg/kg/minute**
MAP (mmHg)	73	60	63	72	78
HR (beat/minute)	130	103	113	110	118
PAOP (mmHg)	14	14	14		
PASP (mmHg)	56	58	66		
PADP (mmHg)	50	43	45		
MPAP (mmHg)	53	48	52		
CI (L/minute/m^2^)	2.04	2.33	2.1	2.5	3.4
SV (mL)	24.5	36.9	30.8	36.3	47
SVRI (dyne·second/(cm^5^·m^2^))	2262	1956	1880		
PVRI (dyne·second/(cm^5^·m^2^))	1525	995	1419		
SvO_2_ (mmHg)	42				67.4
Lactate (mg/dL)	4.2				1.7

*Abbreviations*: CI, cardiac index; HR, heart rate; MAP, mean arterial pressure; MPAP, mean pulmonary artery pressure; PADP, pulmonary artery diastolic pressure; PAOP, pulmonary artery occlusion pressure; PASP, pulmonary artery systolic pressure; PVRI, pulmonary vascular resistance index; SV, stroke volume; SvO_2_, venous oxygen saturation; SVRI, systemic vascular resistance index.

These PAC parameters were the same as observed by Condliffe *et al*. [12] and Murata *et al*. [13] except for the pulmonary vascular resistance index, which was greater in our patient in comparison to the Condliffe *et al*. cases (715 versus 1525 dyne·second/(cm^5^·m^2^)). Because connective tissue disease may cause PAH because of the vasculature pathology, the difference in more severe resistance may be explained by the changes in the rheological properties (1 poise (P) = 1g·(second·cm)^-1^ = 1dyne·second/cm^2^) present in the HVS, where a decrease in the blood flow can explain the resistance increase. Laboratory blood samples hinted about the thickness of blood, so viscosity was measured and found to be 65.8cP (<1.9cP).

Lymphadenopathy and organomegaly were ruled out. A bone marrow biopsy did not show clonality of plasma cells. Serum protein electrophoresis confirmed the presence of a massive polyclonal hypergammaglobulinemia, and no paraproteinemia or monoclonal cell population was found from the electrophoretic pattern of the patient’s plasma. Hypergammaglobulinemia was the cause of HVS associated with autoantibodies. Three sessions of plasma exchange therapy were made. Albumin and frozen fresh plasma were used as the expander, and one circulating blood volume was utilized. Significant mental, hemodynamic recovery and clinical outcomes became evident. A negative Q value was observed in the HVS in this patient after taking into account the Starling vascular permeability formula, where the microvasculature is influenced by protein content and transcapillary fluid flux, specifically, where Q is equal to volume of flow across the capillary wall. This finding led to the conclusion that dilution of protein content may alleviate the HVS, and the fluid administration was considered supportive therapy with judicious consideration of the compartment shift of the solutions. His renal function returned to basal state and he was extubated on day 7 of ICU admission. He was then discharged from the ICU and hospital, respectively with good prognosis (Charlson comorbidity index of 0 points).

The patient now attends external consult without respiratory symptoms. One month after hospital discharge his arthritis returned and methotrexate was resumed with good clinical response. Hydroxychloroquine with steroids were initiated because of persistent elevated anti-double stranded DNA (last value at February 2013, 20.2U/mL), no bleeding sign had been present with persistent positive VWF:Ag values.

## Conclusions

HVS with PAH presentation in a patient with aVWD in an autoimmune context is a rare condition that can be treated successfully with plasmapheresis and critical care support.

## Consent

Written informed consent was obtained from the patient for publication of this case report. A copy of the written consent is available for review by the Editor-in-Chief of this journal.

## Abbreviations

aVWD: Acquired von Willebrand disease; DNA: Deoxyribonucleic acid; ER: Emergency room; HVS: Hyperviscosity syndrome; ICU: Intensive care unit; Ig: Immunoglobulin; PAC: Pulmonary artery catheter; PAH: Pulmonary arterial hypertension; PAI-1: Plasminogen activator inhibitor-1; RA: Rheumatoid arthritis; RCo: Ristocetin cofactor; SLE: Systemic lupus erythematosus; VWF: von Willebrand factor; VWF:Ag: von Willebrand factor antigen.

## Competing interests

The authors declare that they have no competing interests.

## Authors’ contributions

TH-G, ER-S, GD-C and SAÑ-S analyzed and interpreted the patient data regarding the treatment at the ICU. YA-F and AGV-R analyzed and interpreted the patient data regarding rheumatology and hematological diseases. SAÑ-S copyedited the manuscript. All authors read and approved the final manuscript.
